# Thoracic Surgeons' Perception of Frail Behavior in Videos of Standardized Patients

**DOI:** 10.1371/journal.pone.0098654

**Published:** 2014-06-03

**Authors:** Mark K. Ferguson, Katherine Thompson, Megan Huisingh-Scheetz, Jeanne Farnan, Josh A. Hemmerich, Kris Slawinski, Julissa Acevedo, Sang Mee Lee, Marko Rojnica, Stephen Small

**Affiliations:** 1 Department of Surgery, The University of Chicago, Chicago, Illinois, United States of America; 2 Department of Medicine, The University of Chicago, Chicago, Illinois, United States of America; 3 Pritzker School of Medicine, The University of Chicago, Chicago, Illinois, United States of America; 4 Center for Research Informatics, The University of Chicago, Chicago, Illinois, United States of America; 5 Department of Health Studies, The University of Chicago, Chicago, Illinois, United States of America; 6 Department of Anesthesiology and Critical Care, The University of Chicago, Chicago, Illinois, United States of America; University of Louisville, United States of America

## Abstract

**Background:**

Frailty is a predictor of poor outcomes following many types of operations. We measured thoracic surgeons' accuracy in assessing patient frailty using videos of standarized patients demonstrating signs of physical frailty. We compared their performance to that of geriatrics specialists.

**Methods:**

We developed an anchored scale for rating degree of frailty. Reference categories were assigned to 31 videos of standarized patients trained to exhibit five levels of activity ranging from “vigorous” to “frail.” Following an explanation of frailty, thoracic surgeons and geriatrics specialists rated the videos. We evaluated inter-rater agreement and tested differences between ratings and reference categories. The influences of clinical specialty, clinical experience, and self-rated expertise were examined.

**Results:**

Inter-rater rank correlation among all participants was high (Kendall's W 0.85) whereas exact agreement (Fleiss' kappa) was only moderate (0.47). Better inter-rater agreement was demonstrated for videos exhibiting extremes of behavior. Exact agreement was better for thoracic surgeons (n = 32) than geriatrics specialists (n = 9; p = 0.045), whereas rank correlation was similar for both groups. More clinical years of experience and self-reported expertise were not associated with better inter-rater agreement.

**Conclusions:**

Videos of standarized patients exhibiting varying degrees of frailty are rated with internal consistency by thoracic surgeons as accurately as geriatrics specialists when referenced to an anchored scale. Ratings were less consistent for moderate degrees of frailty, suggesting that physicians require training to recognize early frailty. Such videos may be useful in assessing and teaching frailty recognition.

## Introduction

Surgical risk estimation is an acquired skill; whether it can be taught is uncertain. Judgment about the appropriateness of surgery or anticipated postoperative outcomes is influenced by a variety of clinical and nonclinical factors. The risk of major complications after some operations is high, leading to prolonged duration of hospitalization and increased costs for affected patients [1], [2]. Physicians are not very accurate in predicting surgical risk when using written vignettes of actual patients, and the additional knowledge of a risk score based on data from test results contained in the vignette does not importantly improve the accuracy of risk estimation [3]. Physicians appear to rely on limited objective clinical data in making risk estimations; instead, they likely base their judgment on other factors including visual cues.

Frailty, a clinical syndrome marked by decreased physiologic reserve and increased vulnerability to stressors, has recently been identified as an independent predictor of adverse outcomes after a variety of medical and surgical interventions [4]–[9]. The determination of frailty is usually based on specific testing or on an amalgam of clinical factors available in administrative databases [10]–[12]. The contribution of a physician's ability to visually assess a patient's degree of frailty, the “eyeball factor,” has not been evaluated or quantified. It is known that evaluating a patient in person has an important effect on how physicians view a patient's level of illness and/or risk in a clinical setting [13], [14]. Visual identification of patients who are likely to be frail may motivate specific testing to assess the degree of frailty and application of interventions that might be useful in reducing surgical risks.

The current study was designed to assess how accurately thoracic surgeons perceive signs of a patient's physical frailty and how their perceptions compare to those of experienced geriatrics specialists.

## Methods

### Ethics statement

This study was approved by The University of Chicago Institutional Review Board (IRB) and subject consent was waived.

### Creation of videos

A group of physicians and other geriatric specialists (MKF, KT, MH-S, JF, KS, SS), using an iterative process, developed a set of physical characteristics based on Fried's phenotypic criteria (weight loss, exhaustion, weakness, slow gait, low physical activity) [15] that could be portrayed in a short, silent video (Table 1). The physical characteristics chosen to be demonstrated in the videos were weight loss, gait speed, strength, and fatigue. We used standardized patients to use in videos for this study. Standardized patients are healthy individuals who are trained to exhibit symptoms of specific disease states for purposes of training and evaluation. Six standardized patients who were male, Caucasian, and middle-aged were hired to portray differing levels of these physical characteristics of frailty in the videos. They underwent a 15-minute training session outlining the frailty phenotype characteristics to be depicted in the videos. The SPs were provided identical wardrobes in two sizes, one purchased to fit, and the other purchased one size larger to help portray weight loss. Silent video recordings that were supervised (MKF and KS) were made in two sessions, each involving three standardized patients. Each standardized patient was asked to portray levels from “vigorous” to “neither vigorous nor frail” wearing appropriately-fitted clothing, and then portrayed levels from “neither vigorous nor frail” to “frail” wearing oversized clothing. The standardized patient was videotaped walking into a mock examination room, sitting in a metal arm chair next to a desk, rising to his feet, walking 4 feet to an examination table, and climbing onto a step to be seated on the examination table. Each video was assigned to a reference category based on the portion of the frailty scale being portrayed during the videotaping (Table 1).

**Table 1:** Rating system for frailty videos.

Clinical factor	Vigorous	Somewhat vigorous	Neither vigorous nor frail	Somewhat frail	Frail
Weight loss	None	None	Possible – either shirt or pants may appear somewhat loose	Possible – both shirt and pants may appear loose	Noticeable looseness of shirt and pants
Gait	Rapid, purposeful stride	Normal pace, medium stride	Normal pace and stride length	Slow, shortened stride	Very slow, shuffling, shortened stride
Strength	Normal, requires no aids to sit, stand, or climb	Normal, uses one hand for balance when sitting, standing, or climbing onto a step	Normal, uses two hands for balance and aid when sitting and standing; slightly slow to climb onto step and turn	Reduced, some difficulty sitting and standing despite use of two hands; some difficulty in climbing onto and turning on step in order to sit on table	Clearly weak, difficulty sitting and standing, uses upper body considerably in sitting and standing; clear difficulty in climbing onto and turning on step in order to sit on table
Fatigue	None – moves rapidly from chair to table	None – moves without effort from chair to table	None – moves somewhat slowly from chair to table	Mild – some rapid breathing with moderate effort, appears tired, moves slowly from chair to table	Definite – breathless with minimal effort; appears drawn; considerable effort necessary to move from chair to table.

Five videos of one standardized patient were selected for use in anchoring the scale in Table 1, and are available for viewing on-line (Vigorous: https://vimeo.com/52246784; Somewhat vigorous: https://vimeo.com/52251018; Neither vigorous nor frail: https://vimeo.com/52498088; Somewhat frail: https://vimeo.com/52498754; Frail: https://vimeo.com/52498843). Sixty-two video segments of the other five standardized patients were assigned random numbers, and half of the videos were randomly selected for use in the study.

### Subject selection and video scoring

Nine full-time specialists in geriatrics (7 geriatricians, 1 geriatric social worker, 1 geriatric nurse) were recruited to participate through their membership in interest groups in the American Geriatrics Society (http://www.americangeriatrics.org/), and 32 thoracic surgeons were recruited to participate through their membership in the General Thoracic Surgery Club (http://gtsc.org/home/). Study data were collected and managed using the REDCap electronic data capture tool hosted at the University of Chicago. REDCap (Research Electronic Data Capture) is a secure, web-based application designed to support data capture for research studies [16]. Participants provided the number of years of experience since completing training in their profession (dichotomized as ≤5 years or >5 years, arbitrarily selected to differentiate between less and more experienced practitioners) and rated their own skill level for assessing frailty as “learner,” “competent,” or “expert” (definitions were not provided to participants). They reviewed the rating system, including the text anchoring the behaviors to be evaluated, and viewed the anchoring videos. The rating system and accompanying videos were available for reference throughout the remainder of the process. The physicians then viewed and rated all 31 video segments in numerical order of their random numbers using the 5-point anchored scale of “vigorous” to “frail” in Table 1 (data available in Ferguson supporting information file Table S1).

### Data analysis

Exact agreement among raters was assessed with the use of Fleiss' kappa (κ). Kappa values range from -1 to +1. For most purposes values greater than 0.60 represent substantial to almost perfect agreement, less than 0.40 reflect poor agreement, and 0.40 to 0.60 indicate moderate agreement beyond chance [17]. Relative agreement among raters was assessed using Kendall's coefficient of concordance (W), in which rank correlation is measured. Because the video reference values are ordinal, assigning an immediately adjacent value carries more weight than assigning a value that is more disparate to that of another rater. Agreement of raters' values with the reference value for each video was assessed using Kendall's correlation coefficient (τ), another measure of rank correlation. For W and τ, possible values range from 0 to 1. A value of 1 indicates perfect agreement, while a value of 0 indicates agreement that would be expected by chance. For each rating group a percentage of exact agreement with the reference value was also determined. Fleiss' Kappa, Kendall's w and tau were compared according to medical specialty (geriatric specialists, thoracic surgeons), self-determined level of expertise, and years of clinical experience with use of bootstrap method in which 10,000 samples of 41 physicians were randomly selected from the original data with replacement. For each sample, the difference in the statistic (i.e., Fleiss Kappa, Kendall's w and tau) between groups was calculated and its p-value were derived. All analyses were performed using R software version, 3.0.1.

## Results

The physicians had 15.9±11.9 years of experience, 17.3±12.1 for thoracic surgeons and 11.0±10.0 for geriatrics specialists (p = 0.134). Nine of the thoracic surgeons rated their ability to recognize frailty as “expert,” followed by 21 “competent” and 2 “learner.” This compared to 3 of the geriatrics specialists rating their ability as “expert” and 6 as “competent” (p = 0.762). There were no differences among years of clinical experience grouped by self-rated ability to recognize frailty (p = 1.00).

Fleiss' κ (exact matches) for all raters was 0.471, indicating moderate agreement [17], whereas Kendall's W was 0.847, indicating high inter-rater rank correlation (Table 2). Rank correlation relative to reference categories was slightly lower for all raters (Kendall's τ 0.797). Surgeons had higher Fleiss' kappa values than geriatrics specialists (0.491 vs 0.390, respectively; p = 0.0452). Rank correlation for inter-rater agreement was similarly high for each group, as was rank correlation relative to reference categories (Table 2).

**Table 2:** Exact agreement and rank correlation for all raters and for raters according to specialty.

Value	All raters	Surgeons	Geriatricians	p value
	n = 41	n = 32	n = 9	
Fleiss' kappa (κ)	0.471	0.491	0.390	0.0452
Kendall's W	0.847	0.850	0.847	0.909
Kendall's tau (τ)	0.797	0.801	0.782	0.333

Fleiss' kappa: exact inter-rater agreement; Kendall's W: relative agreement among raters; Kendall's tau: relative agreement with a standard value

Scoring patterns of physicians who rated their ability to recognize frailty as “expert” were compared to other raters (“competent,” “learner”). Fleiss' kappa for experts was similar to that for other raters (Table 3). Self-rated experts had higher Kendall's W values than other raters; the difference approached statistical significance. In contrast, Kendall's τ values were similar between the groups. No differences in inter-rater agreement were identified comparing less experienced to more experienced clinicians (Table 3).

**Table 3:** Exact agreement and rank correlation according to expertise and years of experience.

Value	Expert	Other	p value	≤5 years	>5 years	p value
	n = 12	n = 29		n = 32	n = 9	
Fleiss' kappa (κ)	0.444	0.479	0.444	0.460	0.501	0.431
Kendall's W	0.887	0.837	0.0531	0.844	0.876	0.294
Kendall's tau (τ)	0.783	0.802	0.274	0.797	0.796	0.944

Fleiss' kappa: exact inter-rater agreement; Kendall's W: relative agreement among raters; Kendall's tau: relative agreement with a standard value

**Figure 1:**
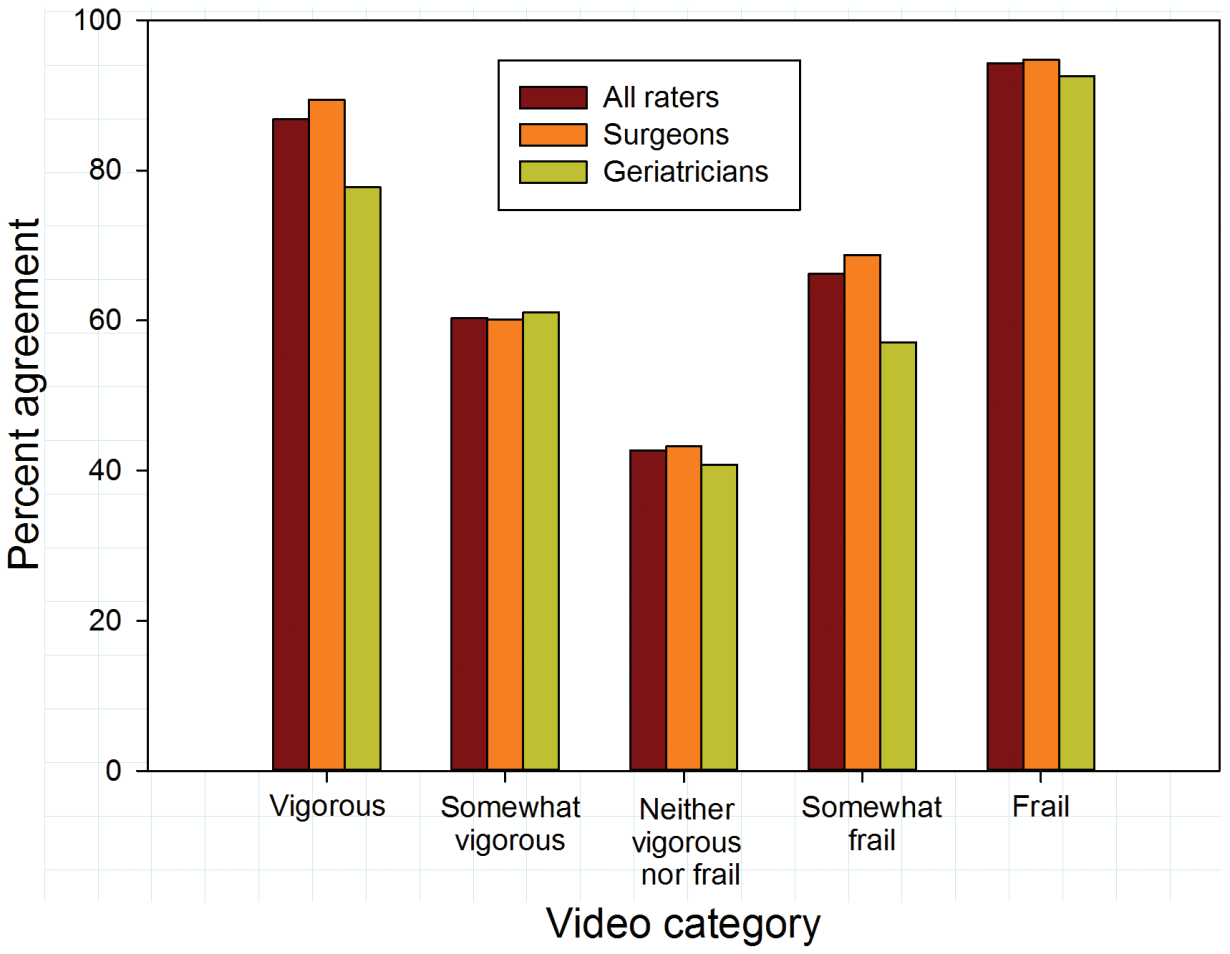
Exact agreement with reference scores by video category for all raters and according to specialty.

**Figure 2:**
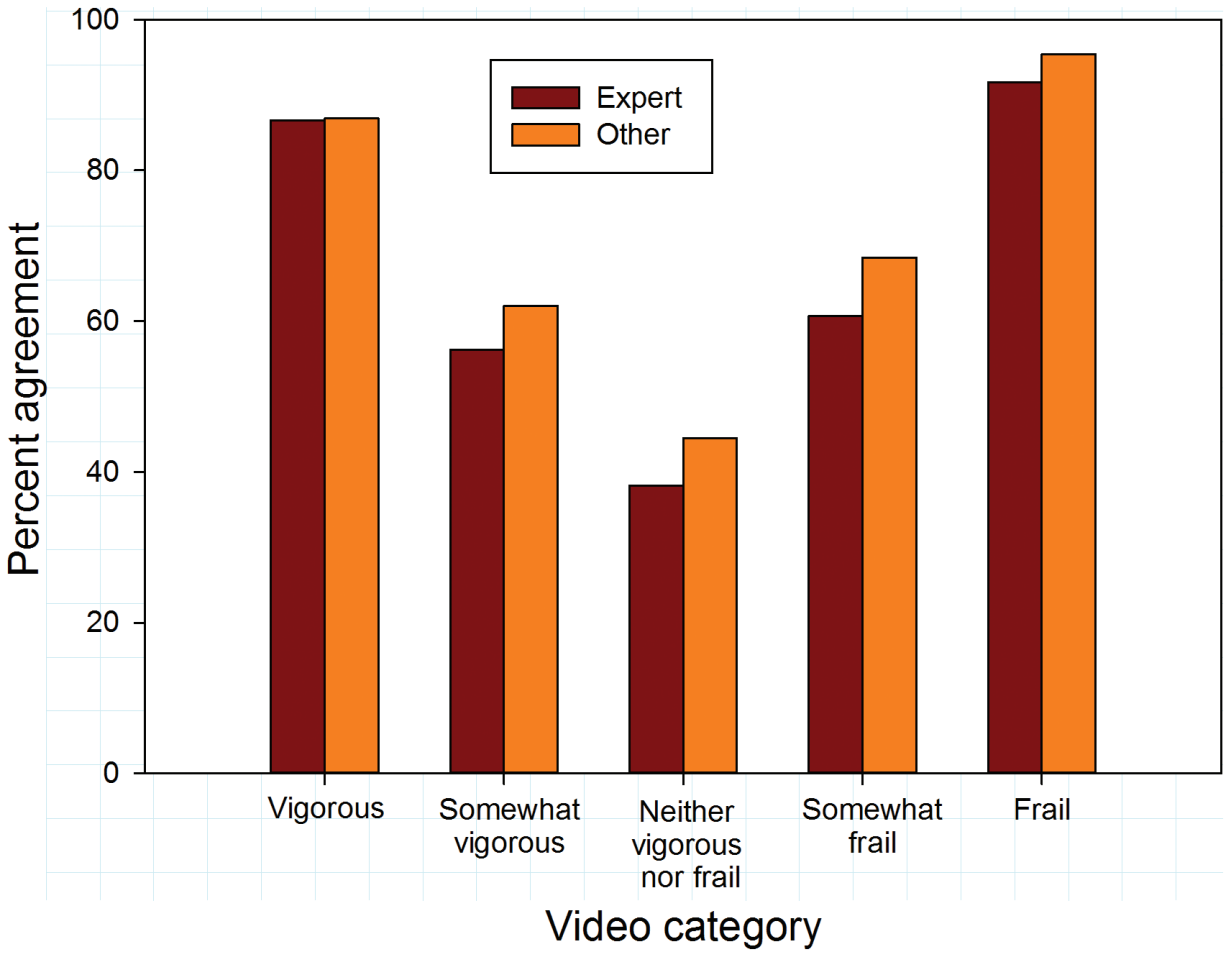
Exact agreement with reference scores by video category according to self-described expertise in recognizing frailty.

**Figure 3:**
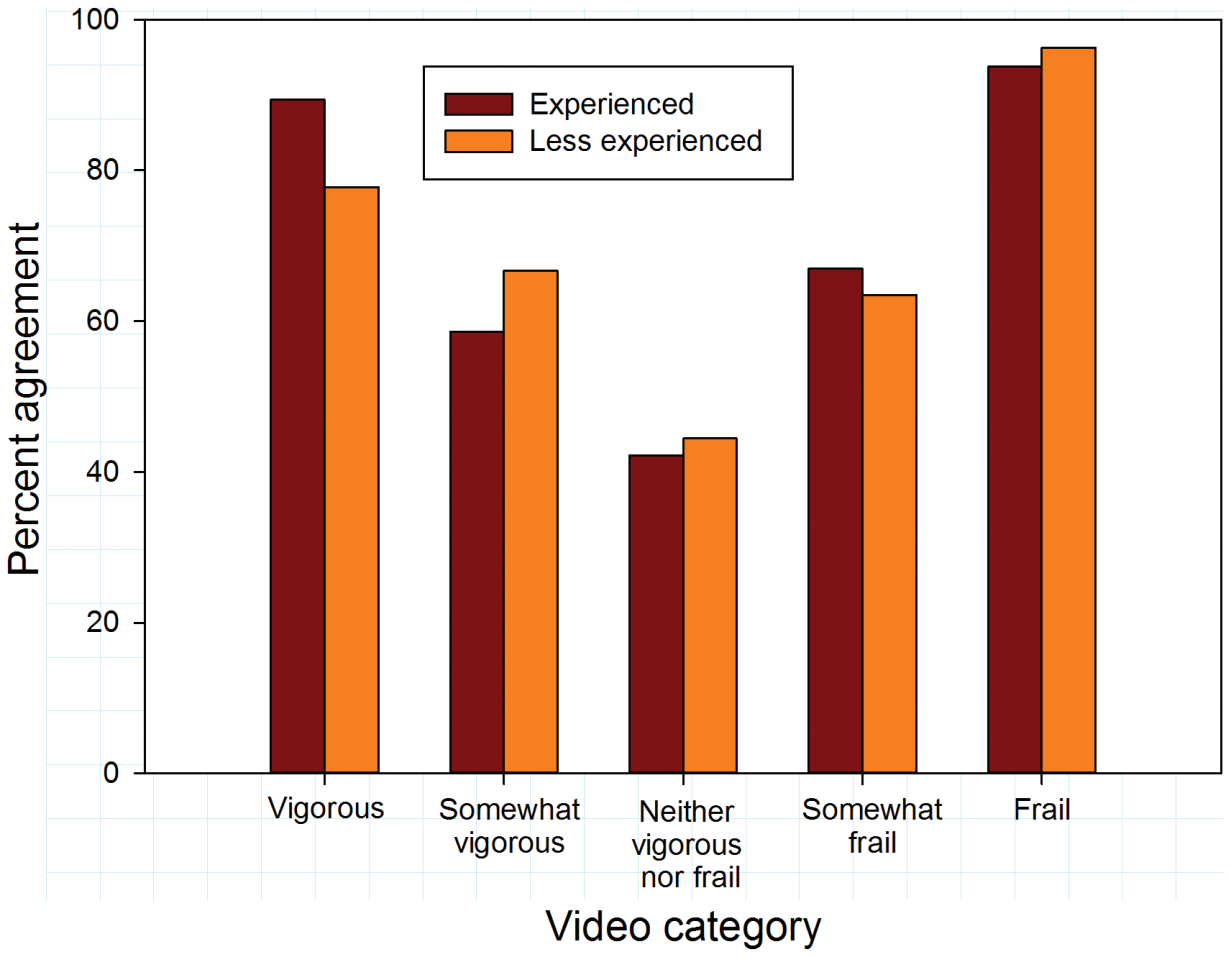
Exact agreement with references scores by video category according to years of clinical experience.

Overall agreement (exact matches to reference values) was somewhat higher for surgeons than for geriatricians (63.6% vs 58.1%; p = 0.090). Video categories importantly influenced inter-rater agreement. Overall agreement was substantially higher for vigorous and frail videos than for videos with intermediate levels of frailty or vigor (Figure 1; p<0.001). Agreement with reference values was higher for surgeons than for geriatricians for vigorous videos (p = 0.042). There were no differences between the specialties in their agreement for other video categories. Similar disparities in agreement rates for videos at the extremes compared to videos portraying intermediate activity levels were evident comparing experts to non-experts and experienced to less experienced raters (Figures 2 and 3).

### Comment

Accurate risk estimation may assist in patient selection for surgery, obtaining informed consent, activating risk reduction protocols for higher risk patients, and determining the need for additional perioperative institutional and community resources. Frailty is increasingly recognized as an important determinant of adverse outcomes and early mortality in a variety of surgical and non-surgical settings. It is associated with poor physiologic reserve, or lack of resilience [18]. Assessment of frailty status is not performed routinely for patients undergoing higher risk operations, and many surgeons and surgical trainees are familiar neither with the frailty syndrome itself nor its impact on surgical outcomes. We sought to determine whether physical signs of frailty portrayed in videos of standardized patients are recognized reliably by thoracic surgeons, and how surgeons' assessments compare to those of experts in geriatric medicine who assess frailty regularly.

We found that participating physicians had moderate inter-rater agreement in exactly matching categories of videos of standardized patients. Surgeons were better than geriatric specialists in exact inter-rater agreement, but the two groups were similar in their rank correlation scores. When relative agreement was assessed using rank correlation, agreement levels were quite high among both surgeons and geriatrics specialists, suggesting that the differences in assigned ratings among participants, when not exact matches, were relatively small. The level of self-rated expertise in recognizing frailty and the number of years of clinical practice did not correspond to the level of inter-rater agreement.

Geriatrics specialists have expertise in recognizing and globally assessing frailty among older adults. Their ratings in this study might have been based more on clinical experience and they might have been less likely to rely on the anchored scale in making their determinations. Surgeons rarely have formal training in frailty recognition and assessment, and were likely to rely primarily on the anchored scale in rating the videos. Successful surgeons tend to have strong visual-spatial ability [19], and this may have resulted in greater accuracy in matching test videos to the anchoring videos. These different strengths resulted in overall similar rating results between the specialist groups.

Physicians had much better agreement in categorizing behavior at the extremes of physical performance compared to their ability to differentiate among intermediate levels of physical performance in the videos. This is not unexpected, as end points on a continuum are typically more easily recognized than more moderate quantities or values. Ease of recognition of extremes of behavior suggests that use of videos representing the outer bounds of a continuum may not be as beneficial as using representations of more nuanced differences in evaluating physician performance. Physicians overall had only fair rates of exact agreement in categorizing intermediate levels of frailty, suggesting that physicians require training to recognize more intermediate stages of frailty or pre-frailty.

The difference in inter-rater agreement associated with different categories of frailty also may be explained by unequal increments/decrements in activity levels that existed among the anchoring and testing videos. Ideally, increments in behavior should be similar between adjacent categories to optimize a rater's ability to distinguish between them. These potential inequalities may result in participants experiencing difficulty differentiating between two similar physical performance categories. Standardizing incremental levels of physical activity, which is just one element of frailty assessment, could have been accomplished using accelerometers, gait pacing, or other metrics of movement during development of the videos for this study. This consideration may be important in the future development of teaching videos for use in frailty recognition training.

The use of videos as an instrument for learning and evaluation in medicine has recent precedents, especially for debriefings after interactions with standardized patients and in providing on-line information and instruction [20]. To our knowledge, such videos have not been used previously in assessing a physician's ability to recognize frailty. Prior to this study it was not known whether physical behaviors taught to standardized patients and captured on video are sufficient for experts and other experienced clinicians to interpret as representing frailty. The very good rank concordance among participants in this study suggests that physicians, regardless of specialty and years of clinical practice, have the ability to recognize different levels of frailty in videos and match them to anchored categories. This suggests that such videos might be useful in the development of educational and evaluative tools. Future studies will need to assess whether frailty identification skills attained through the videos translate to the ability to accurately identify patients classified as frail through formal frailty testing.

Potential shortcomings of this study include the relatively small numbers of clinicians who participated, particularly among geriatrics specialists. Evidence regarding rating consistencies was strong, but should be viewed in the context of the anchored scale outlining frailty scoring. It is not known how clinicians would fare in screening for frailty using videos or in actual clinical situations without the availability of the rating scale for comparison purposes. A host of factors can influence frailty perception, including such things as patient age, specific physical disabilities, sex, and language comprehension—these were purposely excluded from the current study to permit subjects to focus on specific physical behaviors. In addition, our standardized patients were middle-aged rather than elderly, which is the target population for the frailty syndrome. Despite accurate mimicking of the frailty phenotype, the portrayal of physical frailty by our SPs may have lacked verisimilitude. Finally, patient cognition is an essential element in surgical risk assessment. Impaired cognition often accompanies frailty, but cognition could not be judged using the types of videos we created for this study.

Our results make an early methodological step for evaluating competence in frailty screening and for educational purposes. Rapid and accurate visual assessment of patients for frailty is certain to be important in a wide array of surgical specialties [21]. We have yet to determine whether frailty portrayed in the SP videos is interpreted similarly to frailty evident in videos of actual patients. If this concordance is verified, videos such as those developed for this study could be used to measure differences in clinicians' and trainees' abilities to detect frailty. They also could potentially be a tool for educating clinicians and trainees on how to screen patients for signs of frailty and using such information in assessing surgical risk.

In summary, videos of standardized patients portraying differing levels of frailty are accurately rated by surgeons with very good inter-rater consistency. Such videos may be used to assess a surgeon's ability to screen for frailty and could be an effective tool in educating surgeons about how to recognize frailty.

## Supporting Information

Table S1 Video rating data.
(XLSX)
